# A code within the genetic code: codon usage regulates co-translational protein folding

**DOI:** 10.1186/s12964-020-00642-6

**Published:** 2020-09-09

**Authors:** Yi Liu

**Affiliations:** Department of Physiology, ND13.214A, UT Southwestern Medical Center, 5323 Harry Hines Blvd., Dallas, TX 75390-9040 USA

**Keywords:** Codon usage, Translation elongation, Co-translational protein folding, Intrinsically disordered protein

## Abstract

The genetic code is degenerate, and most amino acids are encoded by two to six synonymous codons. Codon usage bias, the preference for certain synonymous codons, is a universal feature of all genomes examined. Synonymous codon mutations were previously thought to be silent; however, a growing body evidence now shows that codon usage regulates protein structure and gene expression through effects on co-translational protein folding, translation efficiency and accuracy, mRNA stability, and transcription. Codon usage regulates the speed of translation elongation, resulting in non-uniform ribosome decoding rates on mRNAs during translation that is adapted to co-translational protein folding process. Biochemical and genetic evidence demonstrate that codon usage plays an important role in regulating protein folding and function in both prokaryotic and eukaryotic organisms. Certain protein structural types are more sensitive than others to the effects of codon usage on protein folding, and predicted intrinsically disordered domains are more prone to misfolding caused by codon usage changes than other domain types. Bioinformatic analyses revealed that gene codon usage correlates with different protein structures in diverse organisms, indicating the existence of a codon usage code for co-translational protein folding. This review focuses on recent literature on the role and mechanism of codon usage in regulating translation kinetics and co-translational protein folding.

**Video abstract**

**Video abstract**

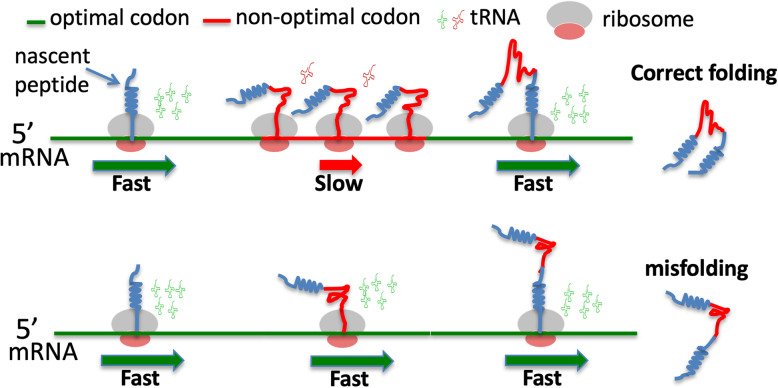

## Background

**Fig. 1:**
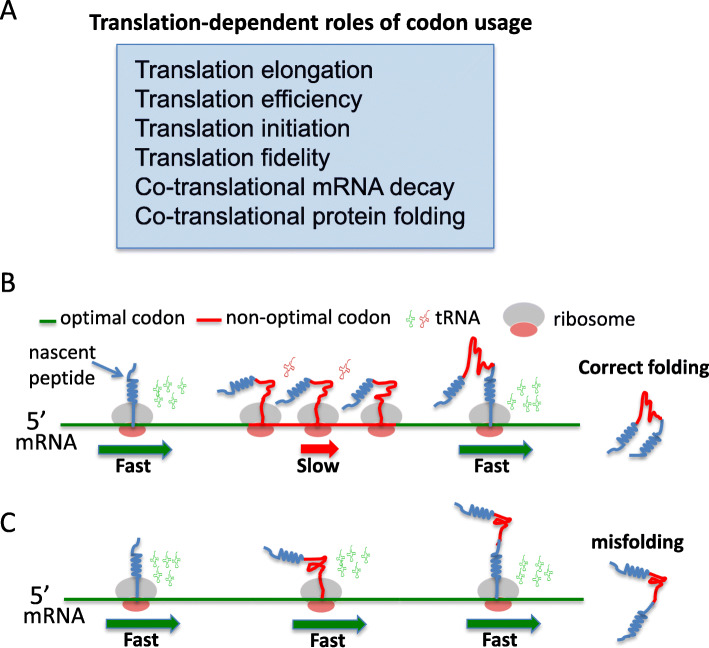
**a** The known translation-dependent roles of codon usage. **b** A model depicting the role of codon usage in regulating translation elongation speed and co-translational protein folding. **c** A model depicting that when codon usage is not optimally adapted to the co-translational protein folding process, protein misfolding increases

Of the 20 standard amino acids, 18 can be encoded by two to six synonymous codons. Preferential use of certain synonymous codons, a phenomenon called codon usage bias, has been found in all genomes evaluated [1–4]. Because synonymous codons encode the same amino acid, they were previously considered to be functionally redundant, and synonymous codon mutations that do not change protein sequences were regarded as silent mutations. However, a large body of evidence now demonstrates that codon usage plays multiple roles regulating gene expression and protein structure through translation-dependent and translation-independent mechanisms [4–8] (Fig. 1a). Synonymous codons are recognized with different efficiencies by cognate tRNAs. In different eukaryotic and prokaryotic organisms, codon usage bias correlates with levels of cognate tRNAs or with tRNA gene copy numbers [1, 9–13]. Codons with strong bias are found to be strongly enriched in highly expressed protein encoding genes, and codon optimization increases endogenous and heterologous gene expression in diverse eukaryotes and prokaryotes [14–23]. Moreover, genome-wide correlations between codon usage bias and protein levels have been observed [17, 24].

Whether codon usage bias can regulate translation kinetics had been the subject of intense debate [25–28], but it is now firmly established that codon usage plays an important role in controlling the speed of translation elongation during mRNA translation [29–32] and that an important role of codon usage bias is to promote translation efficiency [4, 8, 22, 23, 33–35]. Rare codons cause ribosome stalling on an mRNA during translation, which, in eukaryotes, can result in premature translation termination mediated by termination factors [29, 31, 36]. Importantly, codon usage bias also impacts mRNA stability in diverse organisms and can impact on translation fidelity [6, 37–44]. In budding yeast, codon optimality has been shown to regulate the interaction between the mRNA deadenylation complex CCR4-NOT and ribosome during translation due to its effect on translation kinetics, which then affecting co-translational decay of mRNAs [45].

In addition to its translation-dependent roles, codon usage bias has also many translation-independent roles that influence gene expression. In fungal and mammalian cells, codon usage and GC content (which is tightly correlated with codon usage) have been shown to affect mRNA levels by regulating transcription at the level of chromatin structure [17, 23, 46, 47]. Moreover, codon usage can also influence transcription by regulating premature transcription termination and splicing [48, 49]. Therefore, codon usage can influence gene expression through multiple mechanisms at translational, transcriptional and post-transcriptional levels.

In addition to the roles of codon usage in regulating gene expression, a large body of biochemical and bioinformatic evidence has established that co-translational protein folding is influenced by codon usage [5, 13, 20, 29, 50, 51]. These studies led to the proposal that gene codon usage bias represents a new “code” that defines protein structures and protein expression levels. Codon usage regulates the speed of translation elongation, resulting in non-uniform ribosome decoding rates on mRNAs during translation that is adapted to co-translational protein folding. Mutations that do not alter the protein sequence can cause protein structure changes in proteins that are implicated in human disease [23, 52–54]. Thus, codon usage has a potentially underappreciated role in human disease pathogenesis. This review will focus on recent literature on the role and mechanism of codon usage in regulating translation kinetics and co-translational protein folding.

### Codon usage regulates translation elongation speed

Because codon usage biases have been found to correlate with tRNA copy numbers in different organisms, codon usage has been previously proposed to regulate mRNA translation elongate speed because rare codons are hypothesized to take longer to be recognized when their corresponding tRNAs are present at low concentrations [1, 4, 55, 56]. Most studies concerning this issue have relied on indirect measurements and protein overexpression, which led to conflicting conclusions [28, 57–59]. The development of the ribosome profiling technique provided a powerful tool to study ribosome translation dynamics with codon-level resolution in many organisms [26, 60]. Ribosome densities at individual codons should in principle inversely correlate with translation elongation speed; however, early studies based on ribosome profiling data in several organisms found no correlations between codon usage and ribosome density, leading to the conclusion that codon usage does not play a significant role in modulating translation kinetics [25–28].

Because ribosome profiling results could be influenced by experimental conditions, cloning and sequencing biases, methods of bioinformatic analysis, and experimental noise [61–63], we previously re-examined this issue using *Neurospora* and *Drosophila* cell-free translation systems in which translation elongation speed can be directly measured [29, 36]. We measured the time of first appearance of luciferase signal from mRNA templates encoding luciferase with different codon usage profiles (luciferase folding is co-translational in these systems), our results demonstrated clearly that preferred codons speed up rate of translation elongation, whereas rare codons slow translation elongation in both systems (Fig. 1b). This indicated that the effect of codon usage on elongation rate is a phenomenon conserved from fungi to animals. In addition, we found that codon usage controls ribosome traffic on mRNAs and that rare codons result in ribosome pausing and accumulation of nascent peptides of the expected sizes during translation in both amino-acid-context dependent and independent manners [29, 31, 36]. Furthermore, using luciferase-encoding mRNAs with high signal to noise, we convincingly demonstrated the impact of codon usage bias on ribosome occupancy in vitro and in vivo [29]. Using an improved ribosome profiling technique, clear genome-wide correlations between codon usage and ribosome occupancy were also observed [29–32], indicating that the lack of correlation between codon usage and ribosome occupancy in the early ribosome profiling studies was due to the lack of sensitivity and technical issues. Finally, the role of codon usage in translation elongation speed was also confirmed by imaging of single mRNAs during translation in vivo [64]. Together, these studies firmly established a role for codon usage in regulating translation elongation rate and demonstrated that codon usage biases can result in uneven translation elongation speed during mRNA translation (Fig. 1b).

### The role of translation kinetics in protein folding

After synthesis, ordered proteins become functional once they fold properly into stable tertiary structures. Protein misfolding results in impaired protein functions and aggregation or rapid degradation and contributes to many human diseases [65, 66]. Although protein structures are determined by amino acid sequences, it is now widely accepted that folding of most proteins in vivo occurs co-translationally (Fig. 1b). Folding is a modular process that begins on the ribosome as nascent peptide chains are synthesized. Proteins fold from the N-terminal end to the C-terminal end [67–71]. The folding of some protein structure elements, such as α helices, can occur inside the ribosome exit tunnel. Once the newly synthesized nascent chains emerge from the ribosome, their interactions with the ribosome, with protein factors known as chaperones and with other folding catalysts mediate the folding process [67–72]. Translation kinetics was long proposed to influence co-translational protein folding [73, 74]. By using strains with mutant ribosomes that have slow translation speed, by altering tRNA levels, and by growth at low temperature, it was shown that the changes in elongation rate affect folding of proteins overexpressed in *Escherichia coli* [75, 76]. Consistent with this, expression of heterologous proteins often causes protein aggregation due to misfolding, a phenomenon that can be corrected by growth at a low temperature, which presumably slows translation and enhances correct folding.

### The role of codon usage in protein folding

Because codon usage bias often differs throughout a gene coding region, codon usage is expected to result in variation of elongation speed during mRNA translation. As a result, codon usage-influenced translation kinetics should affect the time available for different co-translational folding events. Analyses of in vitro translation and of protein overexpression in *E. coli* cells showed that replacing rare codons with common ones can result in modest decreases of protein activity or solubility [77, 78]. Moreover, synonymous substitutions of common codons with highly abundant tRNAs for codons predicted to slow translation impair the folding of the multidomain protein SufI in vitro and in cells [76]. In addition, using a metric to predict relative translation rates of codons based on tRNA availability, it was shown that synonymous codon harmonization designed to mimic ribosome movement in *E .coli* could significantly increase the specific activity of firefly luciferase overexpressed in *E. coli* [12]. Furthermore, codon usage influences the folding of an artificially designed fluorescent protein expressed in *E. coli* [79]. Importantly, the impact of codon usage on co-translational protein folding was confirmed by monitoring Förster resonance energy transfer in real time and by analysis of fluorescence intensity changes when the mammalian gamma-B crytallin was expressed in *E. coli* [80]. The folding differences due to the presence of different synonymous codon variants was also visualized by NMR spectroscopy and shown to correlate with altered in vivo stability and in vitro protease sensitivity [80]. Recently, analysis of chloramphenicol acetyltransferase with various codon substitution in *E. coli* revealed that codon usage affects protein folding and cell growth [81].

The first suggestion of the role of codon usage in eukaryotic protein folding came from a study that showed that transient expression of variant with a synonymous single-nucleotide polymorphism in the *MDR1* gene, which encodes a protein involved in multidrug resistance, in human cells resulted in altered drug and inhibitor interactions [52]. The genetic role of the codon usage in eukaryotic protein folding was later firmly established by studying genes critical for circadian clock functions in *Neurospora* and *Drosophila* [13, 20, 51]. Unlike most genes in *Neurospora*, the core circadian clock gene *frequency* (*frq)* is enriched for rare codons across its entire open reading frame. Codon optimization of the parts of the *frq* gene with preference for rare codons abolished both overt and molecular circadian rhythms, impaired FRQ activity, and altered FRQ stability and phosphorylation profiles [13, 20]. Such changes are due to changes in FRQ structure, as indicated by altered trypsin sensitivity and resistance to freeze-thaw cycles in vitro. Importantly, the codon optimization of different parts of *frq* can result in opposite molecular phenotypes that can be predicted by local protein strutures, indicating that the codon usage-mediated changes in FRQ structures are due to a local effect of codon usage [20]. Although *frq* and the *Drosophila* circadian clock gene *Period* (*Per*) are not sequence homologs, codon optimization of the parts of *Per* enriched for rare codons also results in abolishment of circadian locomotor rhythm and abnormal molecular rhythmicity due to severe impairment of PER activity in the circadian negative feedback loop and reduction of PER phosphorylation in a location- and codon usage-dependent manner [51]. The changes in PER structure due to codon usage manipulation were demonstrated by altered PER trypsin sensitivity and changes in thermal denaturation and aggregation temperatures. It is important to note that both of the *Neurospora* and *Drosophila* studies did not rely on protein overexpression, and the changes in protein activity and protein structure are not due to protein overexpression as overexpression of the wild-type genes lead to robust circadian rhythms [20, 51]. The roles of codon usage in co-translational protein folding in the *Neurospora* and *Drosophila* are further supported by in vitro translation assays in cell-free translation systems [29, 36]. In addition to the role of codon optimality in determining the speed of translation elongation and translation efficiency of *luciferase* mRNAs, codon usage also affects luciferase folding and activity in vitro and in vivo [29, 36]. Although the codon-optimized *luciferase* mRNA was translated more rapidly and resulted in more full-length protein than wild-type mRNA, the luciferase protein produced was not as functional as the protein synthesized from the wild-type mRNA. These results indicate that when codon usage is not adapted to co-translational folding process, protein misfolding increases (Fig. 1c).

Interestingly, codon optimization of the cyanobacterium *Synechococcus* circadian clock genes *kaiBC* did not impair clock function [19]. On the contrary, the clock function of cyanobacterium *Synechococcus* became more robust upon codon optimization due to an increase in KaiC protein level, which results in decreased cell growth at low temperatures. The different outcomes of circadian clock gene codon optimization in cyanobacterium and eukaryotes suggest that differential structures have different sensitivities to codon usage-mediated co-translation folding (see below).

In human cells, the co-translational folding of the NBD1 domain of the conductance regulator CFTR, which is the transmembrane protein mutated in cystic fibrosis patients, occurs through sequential compaction of different subdomains [54]. The codon optimization of a region of NBD1 domain with non-optimal codon usage increases the protein synthesis rate but also results in aggregation of full-length CFTR, suggesting that translation elongation kinetics of the *CFTR* mRNA are modulated by codon usage in a manner that influences the co-translational folding of different protein regions [54]. In addition, the synonymous single nucleotide polymorphism (SNP) T2562G SNP in the *CFTR* gene introduces a codon recognized by a tRNA that is of low abundance specifically in human bronchial epithelia cells [53]. This SNP leads to changes in CFTR protein stability and channel activity in HeLa cells. Importantly, overexpression of the tRNA that recognizes this rare codon results in wild-type levels of expression, stability, and function of CFTR in HeLa cells, suggesting that alterations in translation elongation kinetics due to the SNP cause impaired CFTR co-translational folding [53]. Codon usage in *KRAS*, which encodes an oncogenic Ras GTPase family member, was shown to regulate the expression and protein structure of KRAS protein when expressed in human cells [22, 23]. More recently, codon usage optimization of the coagulation factor IX and a single SNP of the *ADAMTS13* has been shown to affect protein folding or activity [82, 83]. Together, these studies firmly established the universal role of codon usage in protein folding in both prokaryotes and eukaryotes.

### Predicted intrinsically disordered domain structures are sensitive to codon usage

**Fig. 2:**
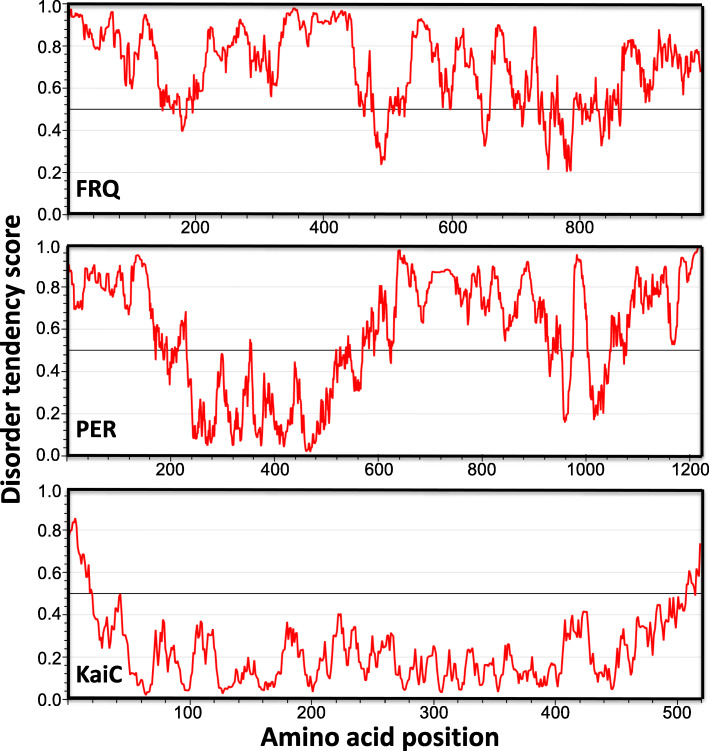
Protein disorder tendency plots of the *Neurospora* FRQ, *Drosophila* PER, and *Synechococcus* KaiC proteins. Protein disorder tendency is predicted by the IUPred2A web interface (https://iupred2a.elte.hu/)

Intrinsically disordered protein (IDP) domains are protein domains that are predicted to lack stable or ordered three-dimensional structures. IDPs have pivotal roles in many biological processes [84–86]. Although IDPs may not form stable structures by themselves, they are often serve as sites critical for post-translational modifications that are important for the overall protein structures and as platforms for inter- or intra-molecular protein-protein interactions [87, 88]. Although both the *Neurospora* and *Drosophila* clock proteins FRQ and PER are large proteins (989 and 1224 amino acids, respectively), most regions of these proteins are predicted to be intrinsically disordered, and these proteins are extensively phosphorylated at many sites along the entire proteins [13, 20, 51, 87, 88] . In contrast, the cyanobacterium *Synechococcus* circadian clock protein KaiC is a highly structured protein for which a high-resolution crystal structure available [89]. The disorder tendency plots of the *Neurospora* FRQ, *Drosophila* PER and *Synechococcus* KaiC are shown in Fig. 2. The opposite circadian phenotypes result from codon optimization of *kaiC* versus *frq* and *Per* suggest that different proteins or protein domains have different sensitivities to codon usage and intrinsically disordered domains are sensitive to change usage changes [13, 19, 20, 51]. This conclusion is further supported by codon optimization of different regions of the *frq* open reading frame: Codon optimization of the IDP regions result in impaired clock functions, whereas codon optimizations of the structured regions have little or no effects on rhythmicity [13].

In the *Neurospora* in vitro translation system, translation of the *luciferase* mRNA with optimal codon usage resulted in markedly reduced luciferase activity compared to the wild-type mRNA despite production of more luciferase protein [29]. By swapping different regions of the *luciferase* mRNA between the wild-type and optimized mRNAs, the region of open reading frame that is most sensitive to codon usage changes was identified [29]. The identified region is highly conserved among luciferase homologs and forms an unstructured loop in the luciferase crystal structure. Thus, both the in vitro and in vivo results suggested that compared to folding of well-structured proteins, co-translational folding of proteins with IDPs is more sensitive to codon usage changes. It is likely that the co-translational folding of IDP-containing domains requires more time and is more sensitive to changes in translation elongation speed than the folding of well-structured domains. Alternatively, fast translation elongation likely interferes with co-translational folding of the structural domains surrounding IDPs.

### Correlations between codon usage and protein structures: the codon usage code for co-translational protein folding

The differential sensitivity of different protein domains to codon usage suggest that protein structures and codon usage has co-evolved to fine-tune the ribosome decoding rates on mRNAs to facilitate the co-translational folding process [19, 29, 74]. Supporting this conclusion, bioinformatics analyses of the relationship between codon usage and protein secondary and tertiary structures uncovered wide-spread correlations [13, 90–97]. Consistent with a role for non-optimal codons in folding of protein domains with IDPs, multiple studies using known structures and predicted secondary structures found that the predicated unstructured and predicted coil domains were found to be enriched for rare codons whereas optimal codons are preferentially found in α-helical regions in proteins from *E. coli*, *Neurospora,* yeast, *C. elegans*, and *Drosophila* [13, 90, 94]. In addition, the translational optimal codons are enriched in buried residues in protein structures, and the association is highest in proteins encoded by highly expressed genes in several organisms [98]. It should be noted that regions with buried residues in highly expressed proteins mostly are found in well-structured protein domains. These bioinformatic studies suggest that well-structured regions are usually encoded by optimal codons whereas unstructured and structurally flexible regions are encoded by rare codons that can induce pauses in the co-translational folding process.

Codon usage also influences other co-translational folding-related processes. Rare codons are enriched in 5′ ends of coding sequences of secreted proteins, and this enrichment was hypothesized to promote membrane targeting and secretion efficiency [99]. In *Saccharomyces cerevisiae*, codon usage in genes encoding some membrane proteins regulates the interaction of nascent polypeptides with the signal recognition particle (SRP), which assists protein translocation across membranes [100]. Co-translational recognition of nascent polypeptides by SRP is enhanced by an elongation pause that is mediated by nonoptimal codon clusters 35 to 40 codons downstream of the SRP-binding site. In human cells, co-translational arginylation of γ-actin, which regulates stability of the protein by influencing ubiquitination, is influenced by rare codons that slow elongation rate in the 5′ end of the gene [101]. In *Aspergillus nidulans*, a pair of rare codons in the gene encoding the urea transporter are important for the production and localization of the UreA protein [102].

Despite the observed correlations, the codon usage to protein structure relationship is a complex one, likely reflecting the complexity of protein folding for diverse protein structures. Although nonoptimal codons were found to be enriched at domain boundaries in some studies [76, 90, 97], a later study failed to find such a correlation and even found a small enrichment of optimal codons [95]. By analyzing homologous coding sequences across different eukaryotic and prokaryotic species, some rare codon clusters were found to be conserved, suggesting a conserved functional role for codon optimality in regulating the folding of homologous proteins [96]. Unexpectedly, the identified conserved rare codon clusters are preferentially located within conserved protein domains. Although the use of different methodologies and different protein structure predictions and databases underlie some apparent contradictions, differences in effects of codon optimization in different protein types and organisms also indicate the complex nature of the co-translational folding processes. The role of codon usage in different proteins and structure types should be dependent on co-translational folding kinetics of different proteins under different cellular environments.

## Conclusions

A growing body of biochemical, genetic, and bioinformatic evidence indicates that the non-uniform decoding rate across mRNAs mediated by codon usage represents a “code” within genetic codons that promote optimal co-translational protein folding process. The adaptation of codon usage to influence the co-translational folding process is the result of evolutionary selection for protein function. Although there are now a few clear genetic examples showing the importance of codon usage in protein folding, more genetic evidence are needed to demonstrate the broad impact of this mechanism in protein folding. The lack of more genetic examples is largely due to the lack of sensitivity of the laboratory assays used. During evolution, even a minute change in protein function that cannot be detected by currently employed assays can have a major effect on organism survival due to the long time frame. Although codon usage influences co-translational protein folding as demonstrated in bacterial and eukaryotic systems, how it facilitates folding of various types of structures is still unclear. Moreover, although synonymous SNPs are associated with many human diseases [103, 104], more future studies will be needed to reveal whether silent SNPs are broad contributors to human disease development due to the role of codon usage in protein structure and gene expression.

## Data Availability

This study does not contain any original data and materials.

## Abbreviations

FRQ: FREQUENCY protein
IDPs: Intrinsically disordered domains
PER: Period protein
SNP: Single nucleotide polymorphism
SRP: Signal recognition particle
